# Effect of Sequence and Stereochemistry Reversal on p53 Peptide Mimicry

**DOI:** 10.1371/journal.pone.0068723

**Published:** 2013-07-29

**Authors:** Alessio Atzori, Audrey E. Baker, Mark Chiu, Richard A. Bryce, Pascal Bonnet

**Affiliations:** 1 School of Pharmacy and Pharmaceutical Sciences, University of Manchester, Manchester, United Kingdom; 2 Biologics Research, Janssen Research and Development Inc., Radnor, Pennsylvania, United States of America; 3 Janssen Research & Development, a division of Janssen Pharmaceutica N.V., Beerse, Belgium; German Research School for Simulation Science, Germany

## Abstract

Peptidomimetics effective in modulating protein-protein interactions and resistant to proteolysis have potential in therapeutic applications. An appealing yet underperforming peptidomimetic strategy is to employ D-amino acids and reversed sequences to mimic a lead peptide conformation, either separately or as the combined retro-inverso peptide. In this work, we examine the conformations of inverse, reverse and retro-inverso peptides of p53(15–29) using implicit solvent molecular dynamics simulation and circular dichroism spectroscopy. In order to obtain converged ensembles for the peptides, we find enhanced sampling is required via the replica exchange molecular dynamics method. From these replica exchange simulations, the D-peptide analogues of p53(15–29) result in a predominantly left-handed helical conformation. When the parent sequence is reversed sequence as either the L-peptide and D-peptide, these peptides display a greater helical propensity, feature reflected by NMR and CD studies in TFE/water solvent. The simulations also indicate that, while approximately similar orientations of the side-chains are possible by the peptide analogues, their ability to mimic the parent peptide is severely compromised by backbone orientation (for D-amino acids) and side-chain orientation (for reversed sequences). A retro-inverso peptide is disadvantaged as a mimic in both aspects, and further chemical modification is required to enable this concept to be used fruitfully in peptidomimetic design. The replica exchange molecular simulation approach adopted here, with its ability to provide detailed conformational insights into modified peptides, has potential as a tool to guide structure-based design of new improved peptidomimetics.

## Introduction

Protein-protein interactions (PPIs) are key to a range of fundamental biological functions. Conversely, erroneous PPIs are linked to pathological conditions such as Alzheimer’s disease, Creutzfeldt-Jakob disease, tumorous conditions and AIDS [1]–[3]. Molecules that can modulate these interactions have potential as therapeutics [4]. However, peptidic ligands are limited by several factors including chemical and conformational stability *in vivo* [5], [6]. Strategies to surmount such issues include the use of D-amino acids and other (non-natural) amino acids, structural restraints and non-peptidic backbones [5], [6].

**Figure 1:**
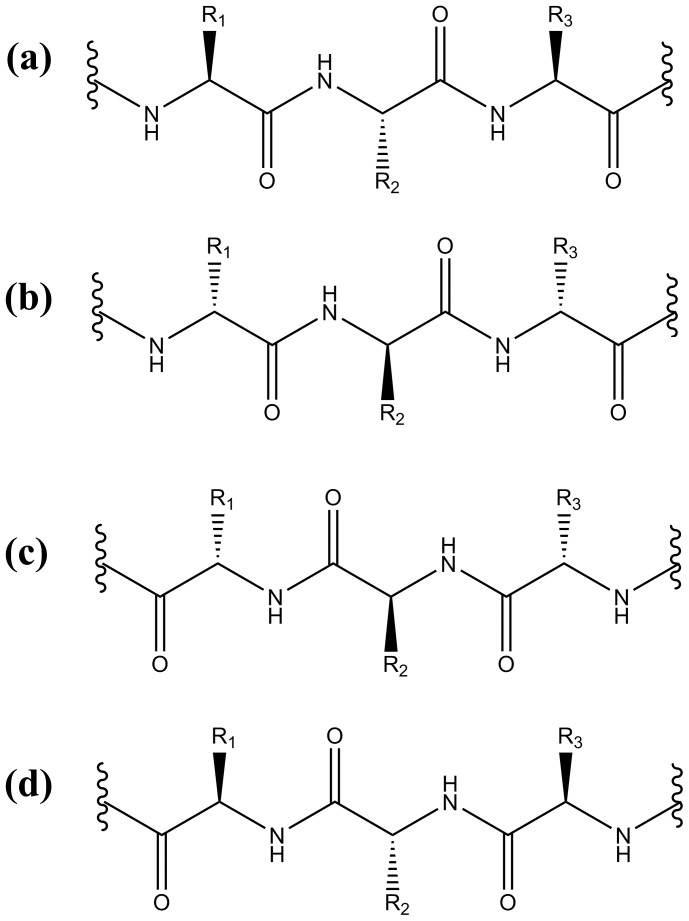
Peptide transformations: (a) parent peptide, (b) its inverse, (c) its sequence reversed and (d) its retro-inverso analogue.

An interesting form of peptidomimetic is the retro-inverso (RI) peptide [7], [8]. The concept is based on two operations: (i) inversion of chirality of the lead peptide (Figure 1a) to produce its enantiomer (Figure 1b) and (ii) reversal of its sequence, which reverses the direction of the termini and peptide bonds. (Figure 1c). When operations (i) and (ii) are combined, a retro-inverso peptide is produced, which should recover the original side-chain orientation of the lead peptide (Figure 1d). This postulate holds for a peptide in its extended form and in helices of the same handedness. The degree of peptidomimicry by RI peptides is less clear when secondary structures are adopted by these and their parent peptides. RI peptides have been shown to elicit cross-reactivity with antibodies against pro-peptide antigens. In perhaps more exacting tests of molecular recognition, however, RI peptides have exhibited mixed success; for example, RI peptides did not perform as well as the corresponding parent peptide in inhibiting the interactions of S-peptide/S-protein [9] and p53/MDM2 [10].

**Figure 2:**
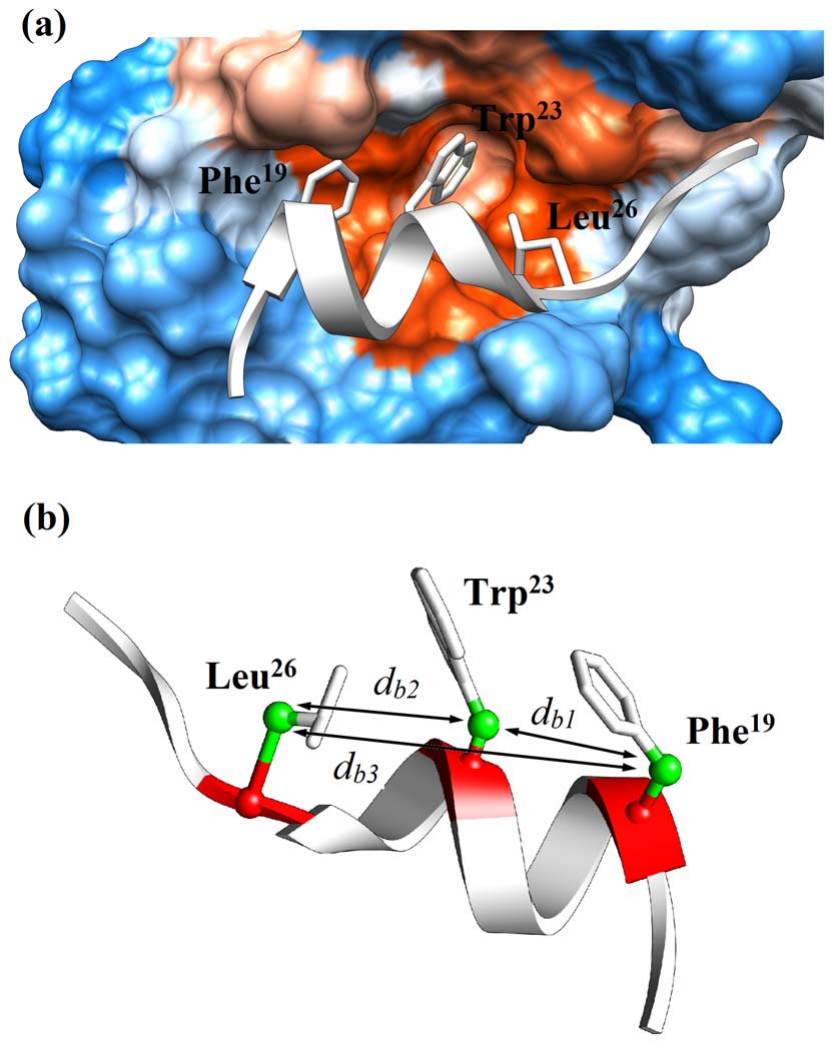
Complex of p53-MDM2: (a) Representation of the N-terminal sequence of p53(white) bound to the MDM2(electrostatic map) derived from its crystal structure. Backbone atoms are represented in cartoon, while the residues Phe^19^, Trp^23^ and Leu^26^ are in sticks. (b) Interatomic distances measured from the crystallographic structure accordingly to the two reference sites, namely α carbon (in red) and β carbon (in green). The three key residues (Phe^19^, Trp^23^ and Leu^26^) are represented in sticks.

The p53/MDM2 is a particularly interesting case, as it highlights the role of secondary structure in determining the inhibitory activity of peptidomimetics. The tumour suppressor p53, a protein of 53 kDa, is a transcription factor involved in cellular response to DNA damage. Considerable interest has focused on the design of modulators of the p53/MDM2 interaction, as inhibition of the human E3 ubiquitin ligase MDM2 leads to reactivation of p53 activity in human glioblastoma cells. The crystal structure of the p53/MDM2 complex indicates that residues 17–29 of p53 interact with a deep hydrophobic cleft on MDM2 [11]. In particular, p53 residues Phe^19^, Trp^23^ and Leu^26^ make extensive interactions with this pocket (Figure 2); to achieve this bound pose, p53 forms a partially α-helical conformation over these residues. Attempts to mimic the p53/MDM2 interaction using mutant p53 peptides [12] show a positive correlation between helicity and inhibitory effect; and peptides stapled into a helical conformation also proved inhibitory [13]. Clearly, secondary structure is important for MDM2 interaction of peptidic ligands, particularly in orienting the key *i*, *i*+4 and *i*+7 side-chains of helical peptides.

The RI peptide of p53(15–29), however, has proved only weakly active against MDM2. Originally, it was postulated by experimental methods that the RI peptide adopted a right-handed helical form [14], contrary to the expected propensity of a D-peptide. Interestingly this harks back to an earlier controversy about the helical handedness of RIs [15]. However, a subsequent NMR structure of the RI of p53(15–29) indicated that a left-handed helix was found in a solution of 50% TFE; no crystals of the RI/MDM2 complex were obtainable [10]. Whilst the concept of retro-inverso peptidomimetics is compelling in its simplicity, to progress their application to structure-based design of protein-protein interactions (PPIs), a greater understanding of their ability to reproduce key structural features of the parent peptide is required. Consequently, in this work, we systematically examine the structural consequences of transformation of the parent p53(15–29) peptide (**WT**, Table 1), considering its stereochemical inverse (**I**), its reversed sequence (**R**) and finally its retro-inverso analogue (**RI**). To achieve these, we first assess the ability of replica exchange molecular dynamics (MD) simulations to reproduce the experimental secondary structural trends of these peptides in aqueous solution that were observed previously [10] and performed here by circular dichroism measurements. To our knowledge, this is the first application of replica exchange MD to characterize D-peptide and retro-inverso conformations. Secondly, we examine the ability of each peptide to mimic the key Phe^19^-Trp^23^-Leu^26^ pharmacophore formed in binding of p53 to MDM2.

**Table 1:** Amino acid sequences and MDM2 binding affinities (K_d_) of peptides p53(15–29) (WT), its inverse (I), its reverse (R) and its retro-inverse (RI); and peptides derived from phage display (^D^PMIα and ^D^PMIβ). D-amino acids residues are indicated with superscript “D”.

*peptide*	*Sequence*	*K_d_ [nM]*
**WT**	Ace-Ser Gln Glu Thr Phe Ser Asp Leu Trp Lys Leu Leu Pro Glu Asn-Nme	255±5 [10]; 140±5 [16]
**I**	Ace-^D^Ser ^D^Gln ^D^Glu ^D^Thr ^D^Phe ^D^Ser ^D^Asp ^D^Leu ^D^Trp ^D^Lys ^D^Leu ^D^Leu ^D^Pro ^D^Glu ^D^Asn-Nme	n/d [16]
**R**	Ace-Asn Glu Pro Leu Leu Lys Trp Leu Asp Ser Phe Thr Glu Gln Ser-Nme	n/d [16]
**RI**	Ace-^D^Asn ^D^Glu ^D^Pro ^D^Leu ^D^Leu ^D^Lys ^D^Trp ^D^Leu ^D^Asp ^D^Ser ^D^Phe ^D^Thr ^D^Glu ^D^Gln ^D^Ser-Nme	71600±8600 [10]
^D^PMIα	^D^Thr ^D^Asn ^D^Trp ^D^Tyr ^D^Ala ^D^Asn ^D^Leu ^D^Glu ^D^Lys ^D^Leu ^D^Leu ^D^Arg	219±11 [17]
^D^PMIβ	^D^Thr ^D^Ala ^D^Trp ^D^Tyr ^D^Ala ^D^Asn ^D^Phe ^D^Glu ^D^Lys ^D^Leu ^D^Leu ^D^Arg	34.5±0.6 [17]

## Results and Discussion

In order to explore via computation the structural consequences of transformation of parent 15-mer p53-derived peptide **WT** into its inverse (**I**), reversed (**R**) and retro-inverso (**RI**) sequences, we first consider the ability of molecular dynamics simulations to provide a converged estimate of their secondary structures. To this end, extended and right-handed helical initial conformations of L-peptides **WT** and **R** were constructed; additionally, left-handed conformations of D-peptides **I** and **RI** were built. The peptides were then simulated for 50 ns in generalised Born implicit solvent using either molecular dynamics or replica exchange molecular dynamics. For REMD, this equates to 3.2 µs of aggregate dynamics per peptide simulation. Good equilibration of the 64 replicas in the REMD simulations across the 270–400 K temperature range was observed (for example, see Supplementary Information, Figure S1 in File S1).

**Figure 3:**
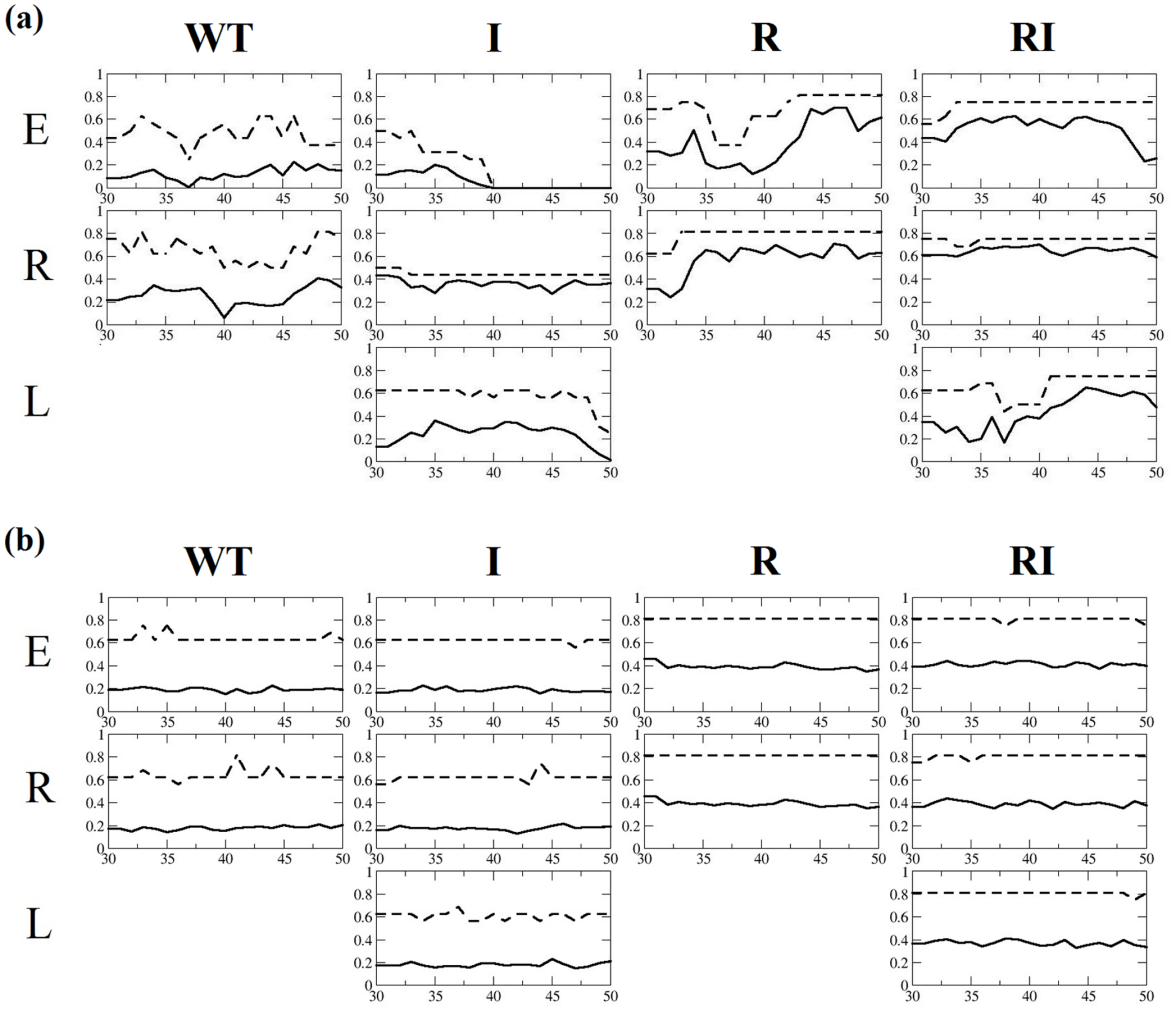
Fractional helical content (ordinate) for sequences WT, I, R and RI as a function of time (ns) (abscissa) obtained from (a) MD simulations and (b) REMD simulations: maximum helicity (dashed line) and average helicity (solid line) obtained starting from extended (E), right-handed (R) and left-handed (L) helical structures.

Over 50 ns of MD, all four sequences fail to establish a converged secondary structure distribution, with a highly uneven estimate of helical content as a function of time (Figure 3a). Likewise, the estimates of helical content vary markedly as a function of initial conformation; this is demonstrated for example by the estimate of 60% helical content for MD starting from the right-handed helix of peptide **RI**, averaged over the last 20 ns, versus 40% helix initiated from an extended conformation (Figure 3a).

**Figure 4:**
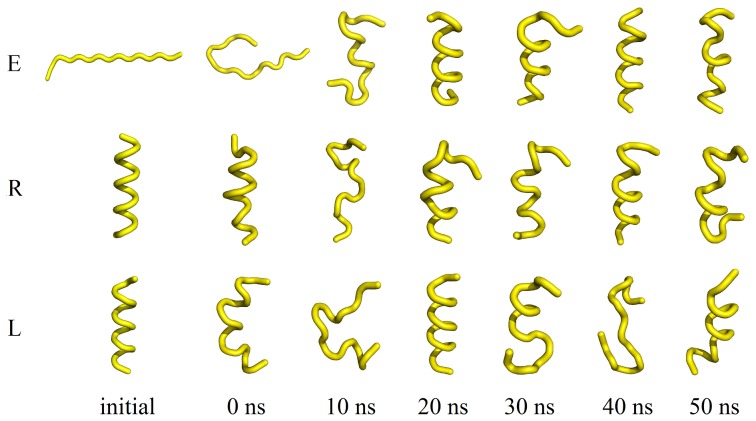
Snapshots of the 300 K trajectory of RI taken at 0, 10, 20, 30, 40 and 50 ns starting from an extended (E), right-handed (R) and left-handed (L) helical structure. Each configuration is ordered from N- to C-terminus.

**Figure 5:**
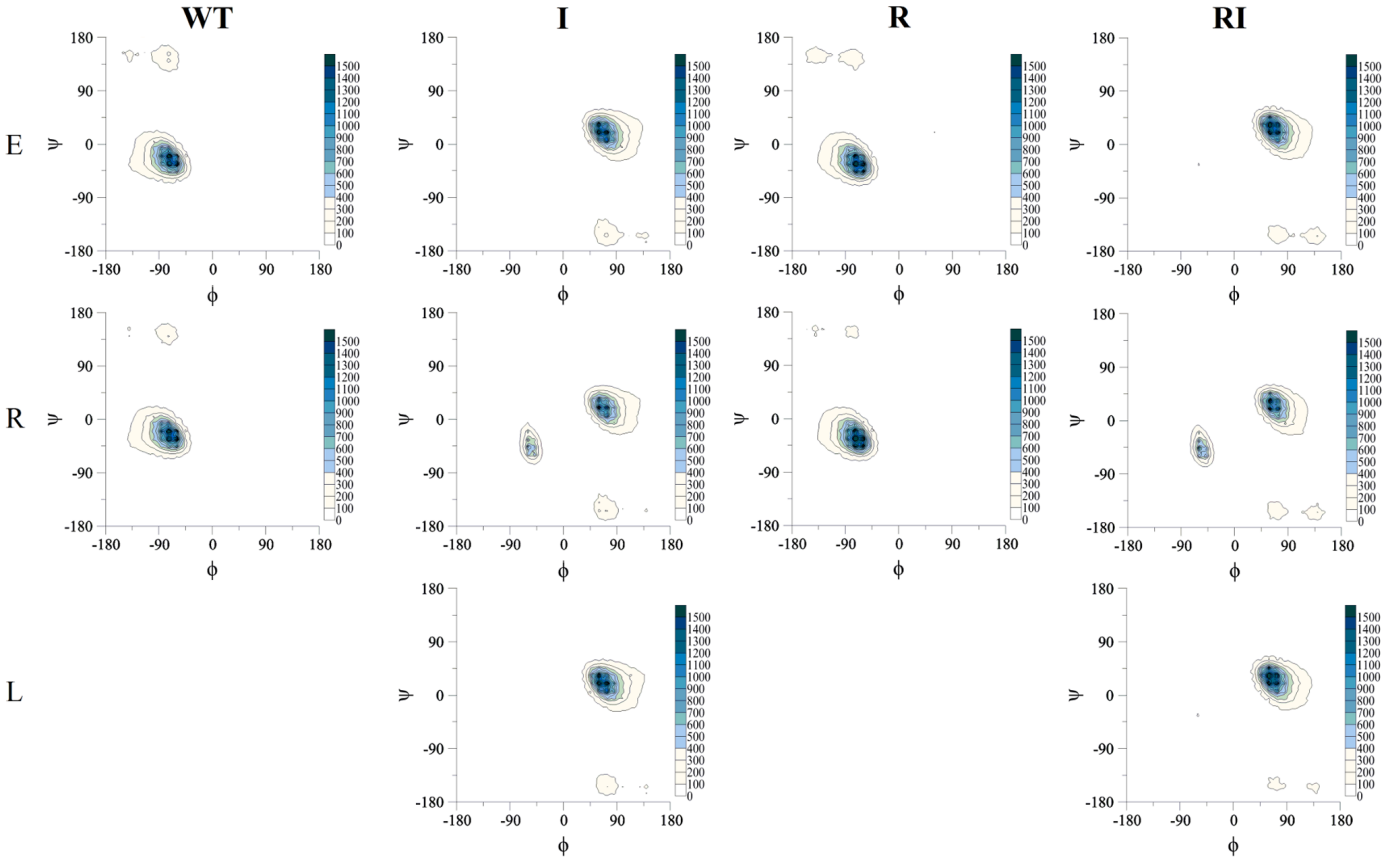
Ramachandran plots of the distribution of the (φ, ψ) backbone dihedrals of residues 1–15 for peptides WT, I, R and RI, calculated over of the 50 ns REMD trajectories.

By contrast, REMD simulations exhibit well-converged estimates of helical content after 30 ns for all four peptides (Figure 3b). Similarly, the helical content is converged between replicate simulations initiated from different conformations (extended, right-handed helix and left-handed helix in Figure 3b). Inspection of snapshots from the time series of the retro-inverso peptide **RI** illustrate this convergence in structure, with extended, left-handed and right-handed helical initial structures each converging over the 50 ns to ensembles containing left-handed rather than right-handed helix (Figure 4). Interestingly these characteristics emerge here after only 20 ns, but it is clear from subsequent structures that an equilibrium of folded and less-folded conformations is established (Figure 4). Nevertheless, over the final 20 ns, the peptides occupy a region around φψ backbone torsion angles of (60°, 30°), in the left-handed helical domain on a Ramachandran plot (Figure 5). The corresponding converged α-helical content for **RI** is computed to be ∼40% (Figure 3b). The Ramachandran plots for peptide **I** similarly indicate convergence to broadly left-handed helical conformations, with a somewhat lower α-helical content of ∼20% (Figures 4 and 5). In addition to hydrogen bonds separated by four residues, the DSSP algorithm also finds a proportion of configurations of **I** and **RI** where *i→i*+3 hydrogen bonds are observed, of ∼18% and ∼11% respectively (Figure S2 in File S1).

By contrast to D-peptides **I** and **RI**, for the 15-mer L-peptides **WT** and **R**, REMD simulations predict a proportion of right-handed rather than left-handed helical structures, with φψ backbone torsion angles converging to the (−60°,−30°) α-helical region (Figure 5). The α-helical content of **WT** reflects its enantiomer **I**, with a value in the vicinity of 20%; likewise, the α-helical content of **R** reflects its enantiomer **RI** (Figure 4). Thus, α-helical content varies with amino acid sequence but, as expected, does not vary between mirror images (this in fact is another measure of the satisfactory convergence of the REMD simulations). Correspondingly, the proportion of 3_10_ helical configurations for **WT** and **R** mirror those of their enantiomers **I** and **RI,** with populations of ∼18% and ∼11% respectively (Figure S2 in File S1). We do also observe a small proportion of conformations in the β-sheet (−60°,140°) region, for both parent peptide **WT** and in a symmetric region (60°,−140°) for its enantiomer **I** (Figure 5). These populations are observed for all replicate simulations of **WT** and **I**; very little of this conformation is found for **R** and **RI**.

We turn now to consider experimental estimates of helicity in these peptides. Circular dichroism measurements have been performed previously [10] for peptides **WT** and **RI**, and indicate that the peptides are largely unstructured in aqueous solution (PBS buffer). Although one must treat absolute values with caution, the helical content estimated from CD in that work was of the order of 10% [10]. To reveal the helical propensity of these peptides, 60% v/v TFE was then used as the helix-promoting solvent. In this solvent, peptide **WT** readily adopted a right-handed helix and **RI** a left-handed helix. Furthermore, the structures of peptides **WT** and **RI** in TFE/water were determined by NMR spectroscopy [10]; although insufficient detail was published to allow detailed comparison with our simulations, **WT** was reported to adopt a flexible structure, which was somewhat disordered at the N- and C-termini but with a helical structure in its centre; peptide **RI** formed a left-handed helix with a more ordered and helical structure. Thus the NMR structures reflect the helicity estimated from CD spectra.

**Figure 6:**
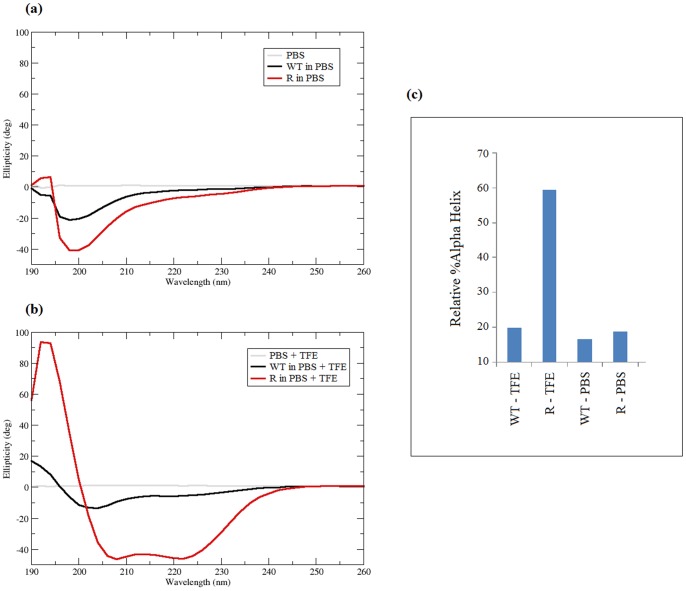
Circular dichroism spectra of peptides: (a) CD spectra of WT and R at 0.3 mg/mL in PBS (b) CD spectra of WT and R at 0.3 mg/mL in 60% TFE/40% PBS (c) percentage peptide α-helix estimated from circular dichroism.

In this work, we have reprised CD measurements to estimate of helicity for **WT** and additionally examine retro-peptide **R**. For spectra obtained in aqueous solution (PBS buffer), the peptides showed strong negative peaks near 195 nm typical of random coil (Figure 6a). The peptides also showed signals near 222 nm typical of helices. The second typical helix signal at 208 nm appears to be masked by the broad peak near 195 nm. The percentage α-helix was obtained from data fitting using CD deconvolution software (see Materials and Methods). The percentage secondary structure element obtained from this software is derived from comparisons of CD structures of globular proteins with known α-helix or β-sheet content. Since the samples examined here are peptides and not globular proteins, there may be some error inherent in the estimation. From this method, the helical contents of **WT** and **R** in PBS buffer were found to be similar: this was around 17% for **WT** and only slightly larger for **R**, at 19% (Figure 6c). When studied in 60% v/v TFE (Figure 6b), peptides **WT** and **R** showed negative peaks at 208 nm and 222 nm typical of alpha helices. Peptide **R** showed the strongest helix signal, while **WT** displayed ∼25% of the intensity seen with **R**: the estimated helical content of **R** was 58% in 60% v/v TFE, whereas that of **WT** was only 20% (Figure 6c). Thus, the mixed TFE/water solvent exposes the greater helical propensity of the reverse sequence, common to **R** and **RI**, relative to the sequence of peptides **WT** and **I**.

This propensity for a larger helical content for **R** and **RI** is reflected in the α-helical populations obtained from our REMD simulations, with values of ∼20% for **WT** and **I**, and ∼40% for **R** and **RI** (Figure 3). The estimates from these aqueous solvent simulations are most comparable with the CD spectra in PBS buffer and therefore are not in quantitative agreement with CD estimates; this discrepancy might depend on a number of factors, including on the algorithm used to compute helicity from the CD spectra (as referred to above), peptide solubility or indeed on the DSSP method used to analyse the simulations. Another factor is the force field and implicit solvent model employed. The *ff99SB* force field used here was derived from *ff99* to improve simulation of secondary structure [18]. We note that an early form of *ff99SB*, combined with the OBC generalized Born solvent model, reproduced explicit solvent simulations of three proteins to within a backbone RMSD of ∼1.5 Å [19]. However, we also note that a recent study of a model tetrapeptide [20], using the same forcefield and solvent model as employed in this work, found, an overstabilisation of ion pair interactions and a greater propensity to form helix relative to explicit solvent simulations, which may indeed be influencing the level of helical content observed in our simulations. We observe that comparable simulations we performed using the *ff96* force field with generalised Born solvent yielded helical content in excess of 80% for peptides **WT** and **RI** (data not shown). Within the limitations of an implicit solvent framework, the simulations based on *ff99SB* provide improved agreement between experiment and calculation, although the predicted helicity remains somewhat higher than estimates obtained from CD.

**Figure 7:**
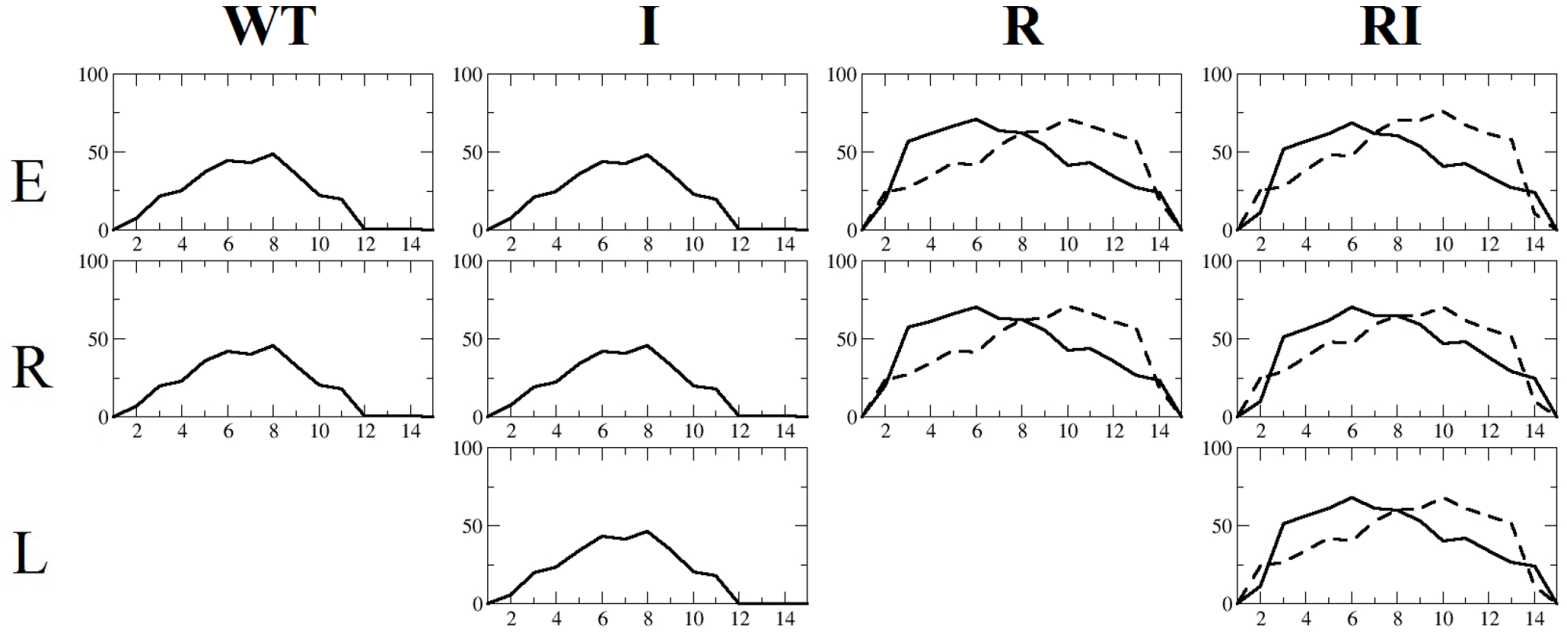
Distribution of helicity (ordinate) as a function of amino acid residue for each of sequences WT, I, R and RI (abscissa), obtained from final 20 ns of REMD simulations with *ff99SB*. Residue numbering is from N- to C-terminal direction (solid line) for **WT**, **I**, **R** and **RI**. Additionally, helicity is shown for the reverse residue numbering ie. from C- to N-terminus for **R** and **RI** (dashed line).

In terms of predicted distribution of helicity across the amino acid sequence of each peptide, our simulations find similar profiles for mirror image pairs **WT** and **I**, and for **R** and **RI** (Figure 7). The higher helicities of **R** and **RI** are evident, extending over the entirety of the peptides and with a peak in helicity around Leu5-Lys6-Trp7 (numbered from the N-terminus). By contrast, peptides **WT** and **I** have lower helicity across the peptide, including at the C- and N-terminii, in qualitative agreement with the NMR structure of **WT** (Figure 7). The peaks in predicted helicity for **WT** and **I** are found around residues Asp7-Leu8-Trp9 (Figure 7), again illustrating the different conformational consequences by sequence reversal.

In order to obtain further insight into the origin of helical preference of reversed sequence (**R**/**RI**) over wildtype (**WT**/**I**), we perform here a detailed analysis of the hydrogen bonding formed within the four peptides over the last 20 ns of REMD simulation. As expected, the total computed fractions of *i→i+4* hydrogen bonds for **R** and **RI** sequences (1.8 and 1.7, Table S1 in File S1) are greater than that of **WT** and **I** sequences (both 0.9). Also, for **WT** and **I** peptides, the distribution of *i→i+4* hydrogen bond population as a function of residue *i* reflects their profiles obtained from the DSSP algorithm (Figure 7), with maxima at residue Leu8 for **WT** and **I** (Table S1 in File S1). For **R** and **RI**, more approximate agreement is found, with maxima predicted at Glu2 and Leu5 (Table S1 in File S1). We note that no hydrogen bond is observed for Trp9– Pro13 in **WT**/**I,** but is observed for Pro3– Trp7 in **R**/**RI** (Table S1 in File S1); in the former, Pro has no backbone NH available to act as proton donor, but in the latter, Pro has a backbone C = O available to act as proton acceptor.

**Figure 8:**
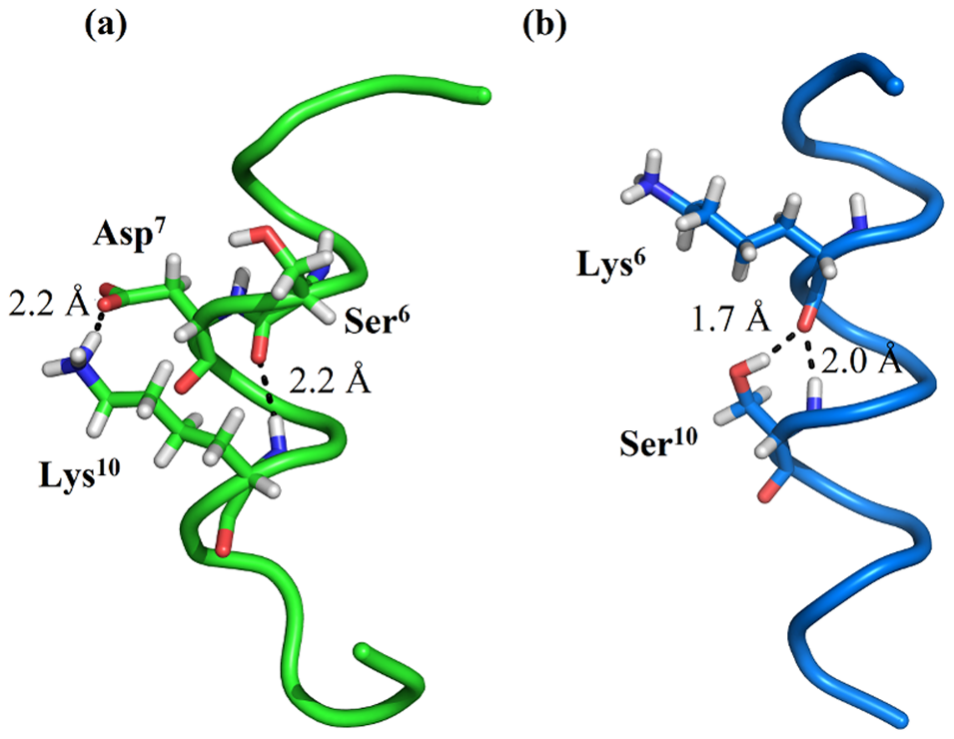
Intramolecular polar contacts. (a) A helical conformation of **WT** when Lys10 NH ^…^Ser6 OC and Lys10 Hζ^…^Asp7 Oδ hydrogen bonds are present; (b) A helical conformation of **R** when Ser10 NH ^…^Lys6 OC and Ser10 Hγ^…^Lys6 O hydrogen bonds are present.

We also examine all hydrogen bonding interactions formed by amino acid side-chains, to other side-chain or main-chain atoms. We find that for **WT** and **I** peptides, only a single hydrogen bond is populated beyond 15% during the REMD trajectories at 300 K; this interaction is a salt-bridge formed between side-chains of Asp7 and Lys10 (Figure 8a), with a population of 20% for both sequences (Table 2). Associated with this interaction, only infrequently is a bridging *i→i+4* hydrogen bond formed between the peptide groups of residues 7 and 11 (<1%, data not shown).

**Table 2:** Population, *p* (%), of most commonly formed intramolecular hydrogen bonds involving side-chains of peptides WT, I, R and RI from last 20 ns of REMD.

sequence	hydrogen bond atom pair	*p*
**WT**	Asp7 Oδ – Lys10 Hζ	20
**I**	Asp7 Oδ – Lys10 Hζ	20
**R**	Lys6 O – Ser10 Hγ	25
**RI**	Lys6 O – Ser10 Hγ	25
**R**	Leu8 O – Thr12 Hγ	22
**RI**	Leu8 O – Thr12 Hγ	21
**R**	Phe11 O – Ser15 Hγ	16
**RI**	Phe11 O – Ser15 Hγ	17

Conversely, for the reverse sequences (**R** and **RI**), three hydrogen bonds are significantly populated (Table 2). In each case, these interactions involve a side-chain hydroxyl group as proton donor (of Ser10, Thr12 and Ser15) and in each case the side-chain OH hydrogen bonds to the corresponding *i-4* residue’s peptide O atom. When this hydrogen bond is made, population of the corresponding α-helical peptide hydrogen bond ranges from 3–10%. A representative MD conformation where the peptide carbonyl O of Lys6 hydrogen bonds to the peptide NH and OH side-chain of Ser10 (Figure 8b) suggests the potentially helix-stabilising nature of these interactions. From inspection, the absence of an analogous interaction in the **WT**/**I** sequence appears to be arise from an increased distance of the side-chain OH to the main-chain O atom.

### Pharmacophore

From the preceding discussion, it is evident that the 50 ns REMD simulations provide a converged estimate of the conformational ensembles of the two L- and two D-peptides in aqueous solution that is in reasonable agreement with experiment. Based on these simulations, we now consider the ability of the right handed helix of sequences **WT** and **R** and left handed helix of sequences **I** and **RI** to mimic the relative spatial locations of the side-chains of the p53 Phe-Trp-Leu triad that is key to its interaction with MDM2 (Figure 2a). We use two measures of relative spatial arrangement: the distance between the α-carbons for Phe-Trp, Trp-Leu and Phe-Leu pairs, denoted *d_a1_*, *d_a2_* and *d_a3_* respectively; and the equivalent inter-β-carbon distances, denoted *d_b1_*, *d_b2_* and *d_b3_* respectively (Figure 2b). From the crystal structure of p53/MDM2 (PDB code: 1YCR [11], resolution 2.6 Å), the distances between Phe^19^ and Trp^23^ and between Trp^23^ and Leu^26^ are in a narrow range of 5.7–6.2 Å over both α- and β-carbon measures (Table 3). Unsurprisingly, the inter-α- and inter-β-carbon distances between Phe^19^ and Leu^26^ are rather larger, at ∼11.7 Å (Table 3).

**Table 3:** Average interatomic distances *d_1_* (Phe^19^-Trp^23^), *d_2_* (Trp^23^-Leu^26^) and *d_3_* (Phe^19^-Leu^26^) as defined by (*a*) α-carbons and (*b*) β-carbons from final 20 ns of REMD for sequences WT, I, R and RI.

Distance/pharmacophore	X-ray distance	MD average distance				population (%)			
	p53(15–29)	WT	I	R	RI	WT	I	R	RI
(a) inter-C_α_									
*d_a1_*	5.89	7.28 (1.45)	7.22 (1.33)	6.99 (1.51)	6.99 (1.43)	31.1	31.2	42.8	42.5
*d_a2_*	5.92	6.02 (1.27)	5.99 (1.29)	5.95 (1.04)	5.96 (0.98)	39.9	39.6	40.1	40.4
*d_a3_*	11.67	11.31 (2.13)	11.31 (1.94)	11.18 (1.89)	11.18 (1.95)	48.4	48.2	40.0	42.4
*[d_a1_-d_a2_-d_a3_]*	–	–	–	–	–	5.0	5.8	4.7	4.7
(b) inter-C_β_									
*d_b1_*	5.67	7.67 (1.81)	7.56 (1.72)	7.38 (1.91)	7.37 (1.83)	20.4	19.8	31.7	29.8
*d_b2_*	6.22	7.04 (1.54)	7.04 (1.49)	6.80 (1.34)	6.80 (1.28)	35.5	36.4	43.1	42.0
*d_b3_*	11.62	11.50 (2.47)	11.56 (2.28)	11.45 (2.48)	11.49 (2.48)	50.9	52.1	43.4	47.4
*[d_b1_-d_b2_-d_b3_]*	–	–	–	–	–	1.5	1.7	1.6	2.1

Standard deviations in parentheses. The *d_1_*, *d_2_* and *d_3_* values from crystallographic structure of N-terminal sequence of p53 bound to MDM2 also given (PDB 1YCR [11]). All distances in Å. Population (%) of trajectory of **WT, I, R** and **RI** that satisfy *d_1_*, *d_2_* and *d_3_* and their simultaneous combination, *[d_1_-d_2_-d_3_]* over final 20 ns of REMD.

We therefore take these two sets of crystallographic distances, defined by either α- or β-carbons, to represent pharmacophores for interaction with MDM2. These are denoted [*d_a1_*–*d_a2_*–*d_a3_*] and [*d_b1_*–*d_b2_*–*d_b3_*] respectively, and correspond to a conformation of the peptide such that all three distances are simultaneously satisfied to within ±0.5 Å of the crystallographic values of the p53 motif when MDM2-bound. Thus, we analyse the last 20 ns of the REMD simulations of **WT**, **I**, **R** and **RI**, to consider what if any population of [*d_a1_*–*d_a2_*–*d_a3_*] and [*d_b1_*–*d_b2_*–*d_b3_*] pharmacophoric motifs are present in the solution ensembles. Peptide **WT**, being directly derived from the p53 sequence, might be expected to exhibit the most highly populated pharmacophore. For **WT,** we find a population of 5.0% of pharmacophore [*d_a1_*–*d_a2_*–*d_a3_*] (Table 3)**.** This fairly low population reflects for example that the average simulated distance *d_1_* is larger than the crystallographic value of *d_1_*, by 1.4 and 2.0 Å for α- and β-carbon definitions respectively (Table 3). Each of the single interatomic distances considered also exhibit significant standard deviations around their mean values (1.5–2.5 Å for inter-C_β_ distances in **WT**), reflecting the dynamic nature of the peptide. With the perhaps more exacting and accurate measure of the three inter-β-carbon distances, the [*d_b1_*–*d_b2_*–*d_b3_*] pharmacophore in peptide **WT** is only populated for 1.5% of the 300 K REMD trajectory. This is set against higher populations of individual distances (20–51%, Table 3). Compared to **WT**, a very similar pattern of populations is observed for mirror image peptide **I**, once again indicating convergence of sampling (Table 3).

For reversed sequence **R** and its mirror image **RI**, the population of pharmacophore [*d_a1_*–*d_a2_*–*d_a3_*] is 4.7% (Table 3). This value is similar to that of **WT** and **I**. The population for **R** and **RI** is also similar to **WT** and **I** when considering [*d_b1_*–*d_b2_*–*d_b3_*], at 1.6% for **R** and 2.1% for **RI** respectively. We note in all cases that there is a similar level of flexibility in the peptides, as reflected by the standard deviation in *d_b1_*, *d_b2_* and *d_b3_* distances ranging from 1.3–2.5 Å for **R** and **RI** (Table 3).

**Figure 9:**
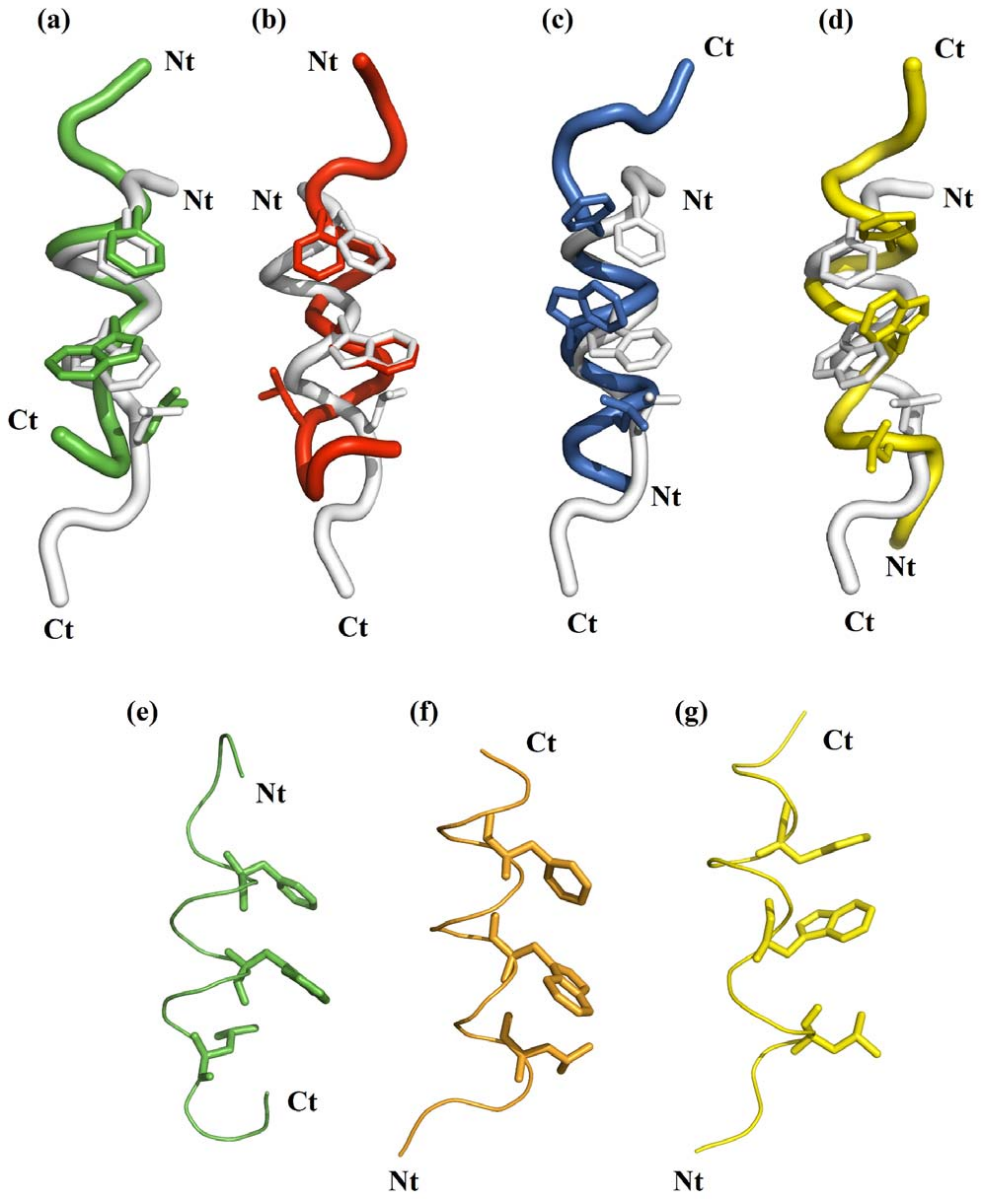
Comparison of peptide conformations. *(top)* Superimposition of C_α_ atoms of X-ray structure of N-terminal sequence of p53 (white) onto representative MD conformations of **WT** (green, a), **I** (red, b), **R** (blue, c) and **RI** (yellow, d). *(bottom)* Comparison of the orientation of the side-chains of the residues Phe, Trp and Leu for the right-handed helix of **WT** (green, e), right handed helix of **RI** (orange, f) and left handed helix of **RI** (yellow, g). Key residues Phe, Trp and Leu represented as sticks.

Consequently, given the similar population of rudimentary pharmacophores [*d_a1_*–*d_a2_*–*d_a3_*] and [*d_b1_*–*d_b2_*–*d_b3_*] by the four peptides, one might expect the retro peptide and the retro-inverso peptide to be quite as capable of interaction with MDM2 as parent p53(15–29) peptide. For further insight, we compare the crystallographic conformation of p53(15–29) with representative MD configurations of **WT**, **I**, **R** and **RI** that exhibit pharmacophore. To achieve this superposition, reversed peptides **R** and **RI** were superimposed in their C*t*→N*t* orientation on the crystal structure in its N*t*→C*t* direction (Figure 9a–d). The side-chains of Phe^19^, Trp^23^ and Leu^26^ in the crystal structure overlap well with those found for peptide **WT** and, to a lesser extent, **I** (Figure 9a, b).

By comparison, whilst the side-chains of **R** and **RI** exist in the same approximate location as for the crystal structure, there is essentially no overlap of the actual side-chain moieties of Phe, Trp and Leu (aromatic rings and isopropyl group). The reversed sequence **R** orients the the C_α_-C_β_ bond vectors, and thus residue side-chains, in opposite quite distinct direction to p53(15–29) when **R** is aligned C*t*→N*t* against p53(15–29) in a N*t*→C*t* direction (Figure 9c). Recovery of a similar orientation for these residues would be achieved by inversion of the reverse sequence only if **RI** retained a right-handed helix; indeed, a modelled hypothetical right-handed helix of **RI** (Figure 9f) demonstrates a similar position of the side-chains to that of the crystal structure and peptide **WT** (Figure 9e), contrasting with left-handed **RI** (Figure 9g). Indeed, experiment and simulation finds that **RI** adopts a left-handed rather than right-handed helix and therefore the side-chains point into volumes quite disparate from the native structure (Figure 9d).

Alongside the differing side-chain orientations of **R** and **RI**, the sense of the left-handed helix dictates a very different volume occupied by the D-peptide backbones of peptides **I** and **RI** (Figures 9b,d); this lack of mimicry could present a potential problem in binding. It should also be borne in mind that the peptide bonds are reversed in peptides **R** and **RI**, which could affect peptide-receptor hydrogen bonds involving these atoms. In summary, whilst helical structures are adopted by **I**, **R** and **RI**, and approximate pharmacophores [*d_a1_*–*d_a2_*–*d_a3_*] and [*d_b1_*–*d_b2_*–*d_b3_*] are fractionally populated, the detailed molecular structures of **I** and **RI** do not suggest strong analogy to parent sequence **WT**. Indeed, retro-inverso peptide **RI** is found to be a weak MDM2 binder experimentally, with a K_d_ of 72 µM, as compared to the sub-micromolar potency of **WT** (Table 1). Although aspects of their findings have been disputed (including their observed high activity of **RI**), Sakurai et al. [14] found no observable MDM2 inhibitory activity for peptides **I** and **R** using an ELISA-based assay.

### Conclusions

In this study, we have examined the consequences of reversed sequence and inverted stereochemistry on structural features of a p53-derived peptide in aqueous solution using molecular dynamics simulations. As a prerequisite to this analysis, we find that, in order to obtain converged ensembles for the peptides, enhanced sampling is required via the replica exchange molecular dynamics method. From these replica exchange simulations, D-peptides **I** and **RI** result in a predominantly left-handed helical conformation, regardless of initial structure. Interestingly, when the parent sequence is reversed to give the L-peptide **R** and D-peptide **RI**, higher helical content with an altered residue profile, is observed for the peptides; this propensity for increased helicity of the reversed sequence is suggested by NMR and CD studies in TFE/water solvent. However the predicted degree of helicity across the four peptides from our simulations in aqueous solution, based on the *ff99SB*/generalized Born potential, appears somewhat high. From the simulations, the increased predicted helicity of the reversed sequences relative to wildtype appears to stem, at least in part, from stabilizing side-chain hydrogen bonding by Ser10, Thr12 and Ser15 with their corresponding *i-4* peptide group; this interaction appears to be precluded in the wildtype sequences, and, from inspection, may be due to a combination of the altered orientation of the C_α_-C_β_ bond vector and an increased distance of the side-chain OH to the main-chain O atom in the wildtype sequence.

From these simulations, we also consider the potential of these modifications to achieve mimicry of the natural peptide. We find that the inter-C_α_ distances or inter-C_β_ distances of the key triad of amino acids are similar between **WT** and its mimics **I**, **R** and **RI**. However, analysis of molecular orientation underlines problems in their alignment of backbone (peptides **I** and **RI**) and their side-chains (peptides **R**, **RI** and to some extent, **I** also). Thus, a retro-inverso peptide is disadvantaged as a mimic in both aspects. That it can produce antigenic mimicry appears a function of the less discriminatory molecular recognition by immunoglobulins. Nevertheless, the concept of using D-peptide mimetics has been successfully utilized in generating inhibitors of MDM2. D-peptides ^D^PMI-α and ^D^PMI-β (Table 1), generated by mirror-image phage display and native chemical ligation, were found to be sub-micromolar inhibitors of MDM2 [17]. Interestingly, crystallography revealed that ^D^PMI-α binds to MDM2 in a left-handed helical conformation, in a pose shifted from p53(15–29). This suggests some flexibility in the backbone (and side-chain) motifs recognized by MDM2. Stapling strategies have been used with success in targeting a number of PPIs using peptides; one could envisage future structure-based drug design strategies employing a retro-inverso peptide which is stapled into a right-handed helical conformation (consider Figures 9e and 9f), thus preserving the conformation of backbone and side-chain whilst benefitting from greater resistance to proteolysis and improved *in vivo* stability. Indeed, the simulation-based approach adopted here, in particular employing replica exchange molecular dynamics, has provided insights into D-peptide conformations and may prove a useful tool in directing the future design of such peptidomimetic structures.

## Materials and Methods

### Computational Details

Four systems were considered: p53(15–29) (**WT**) and its inverse (**I**), reversed (**R**) and retro-inverso (**RI**) sequence (Table 1). Each peptide was capped by an acetyl group (Ace) at the N-terminus and an N-methylamine group (Nme) at the C-terminus. In subsequent discussion, we refer to the residue number 1 to 15 of each peptide; the Ace capping group of the N-terminus is considered residue 0. For each, extended (**E**) and right helical (**R**) conformations were built. For D-peptides, an additional left helical (**L**) form was modelled. The extended structures of the studied sequences were built using XLEAP program from the AMBER 11 [21] suite, whilst right- and left-handed helical structures were created using MOE [22].

The SHAKE algorithm constrained bonds between hydrogen and heavy atoms. A 2 fs time step was used. The peptides were modelled with the AMBER *ff99SB* [18] force field using generalized Born (GB) implicit solvent [19] with a dielectric constant of 80. Simulations were performed in the canonical ensemble NVT with a Langevin thermostat, using a collision frequency of 2 ps^−1^. An infinite cut-off for long-range non-bonded interactions was used [23]. All peptide sequences were minimized prior to 200 ps equilibration, to the desired temperature for each replica in the case of the REMD [24] simulations, or to 300 K for standard MD simulations. For REMD of each peptide, 64 replicas were simulated for 50 ns, using a temperature range from 270 to 400 K. The algorithm of van der Spoel and Patrikkson [25] was used to generate a set of temperatures with the target exchange acceptance ratio of 30%. Exchanges were attempted every ps. REMD and MD simulations were performed using the AMBER 11 [21] molecular simulation package. Configurations were archived every 5 ps.

Secondary structure analysis used the DSSP method by Kabsch and Sander [26] as implemented in the *ptraj* module of AMBER 11, which bases its approach mainly on hydrogen bonding patterns [21]. Calculations of α-helicity were obtained performing by block averaging of the trajectories.

### Circular Dichroism Spectroscopy

CD analyses were conducted using published protocols with slight modifications [27], [28]. For CD analyses, peptides were diluted from stocks at 4 mg/mL in PBS, except sequence **R** which was prepared at 1 mg/mL due to its poor solubility at 4 mg/mL. CD spectra were collected on 0.3 mg/mL peptide solutions in PBS or in 60% (v/v) TFE, 40% (v/v) 1x Dulbecco’s PBS by running four scans at 1 nm intervals from 190 nm –260 nm, at 298 K with 1 sec signal averaging and using 100 ms time constant. Spectra of the PBS and PBS-60%TFE buffers were also collected using the same parameters. Peptide and buffer spectra were averaged and represented as plots of helicity versus wavelength. α-helical content was estimated using CD deconvolution software [29].

## Supporting Information

File S1 Figure S1. Replica exchange equilibration for the (initially) 270 K replica. Figure S2. 3_10_ helical content from REMD simulations of **WT**, **I**, **R** and **RI**. Table S1. Average number of hydrogen bonds between the backbone peptide C = O of residues *i* and the backbone peptide NH of the residues *i +4* and average total number of hydrogen bonds within sequence over final 20 ns of REMD for sequences **WT**, **I**, **R** and **RI**. Standard deviations in parentheses.
(DOCX)
