# Supplementary material for: Effect of Sequence and Stereochemistry Reversal on p53 Peptide Mimicry
Source: PLoS One. 2013 Jul 29;8(7):e68723. doi: 10.1371/journal.pone.0068723 (PMC3726663; doi:10.1371/journal.pone.0068723)
Supplement: File S1 — Figure S1. Replica exchange equilibration for the (initially) 270 K replica. Figure S2. 310 helical content from REMD simulations of WT, I, R and RI. Table S1. Average number of hydrogen bonds between the backbone peptide C = O of residues i and the backbone peptide NH of the residues i +4 and average total number of hydrogen bonds within sequence over final 20 ns of REMD for sequences WT, I, R and RI. Standard deviations in parentheses. (DOCX) [file pone.0068723.s001.docx]

**Supporting Information**

**Effect of sequence and stereochemistry reversal on p53 peptide mimicry**

Alessio Atzori, Audrey E. Baker, Mark Chiu, Richard A. Bryce* and Pascal Bonnet*

*School of Pharmacy and Pharmaceutical Sciences, University of Manchester, Oxford Road, Manchester, M13 9PT, UK*

*Biologics Research, Janssen Research and Development Inc., 145 King of Prussia Road, Radnor, PA, USA*

*Janssen Research & Development, a division of Janssen Pharmaceutica N.V., Turnhoutseweg 30, 2340 Beerse, Belgium*

**Figure S1.** Replica exchange equilibration for the (initially) 270 K replica.


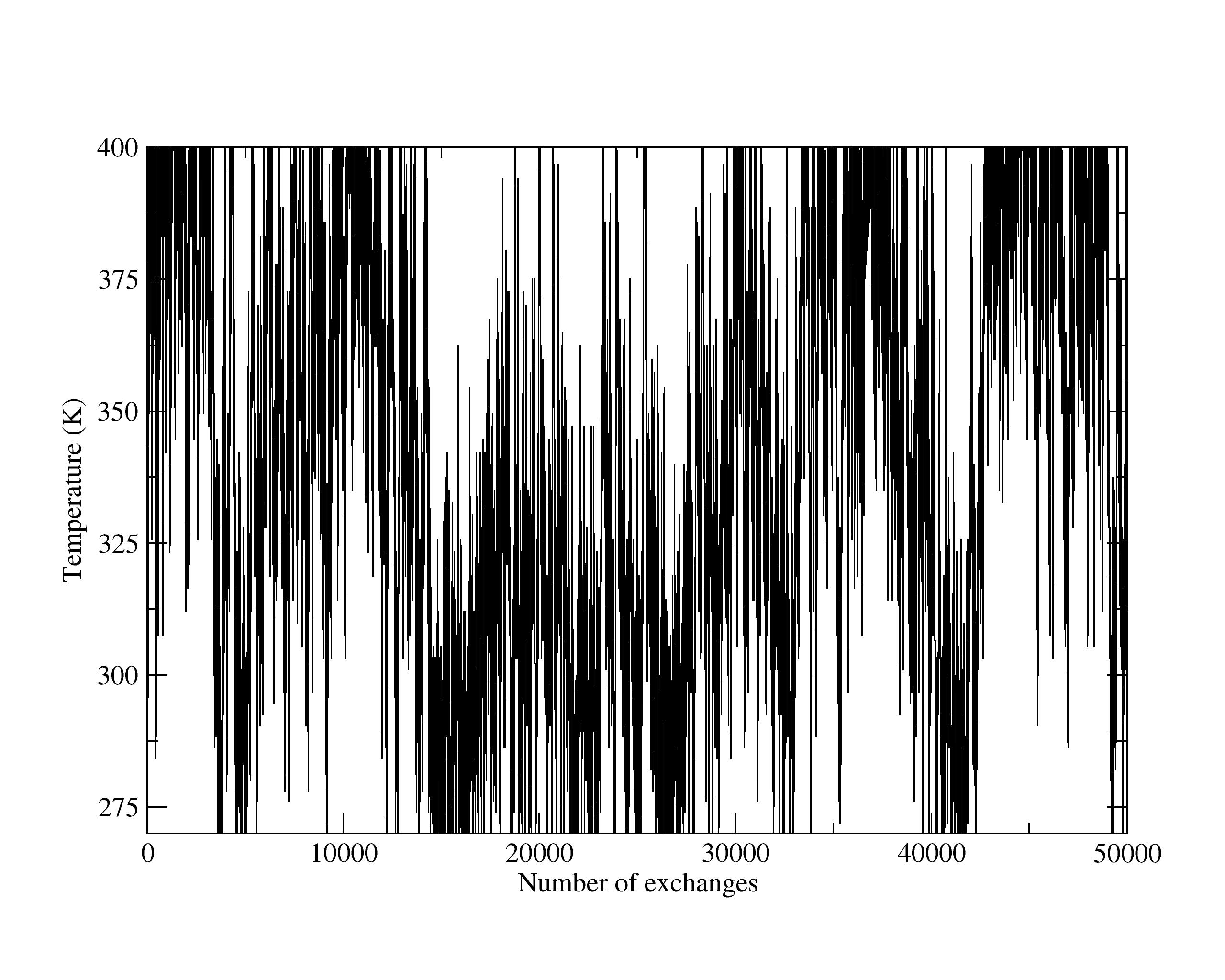


**Figure S2.** 3_10_ helical content from REMD simulations of **WT**, **I**, **R** and **RI**.


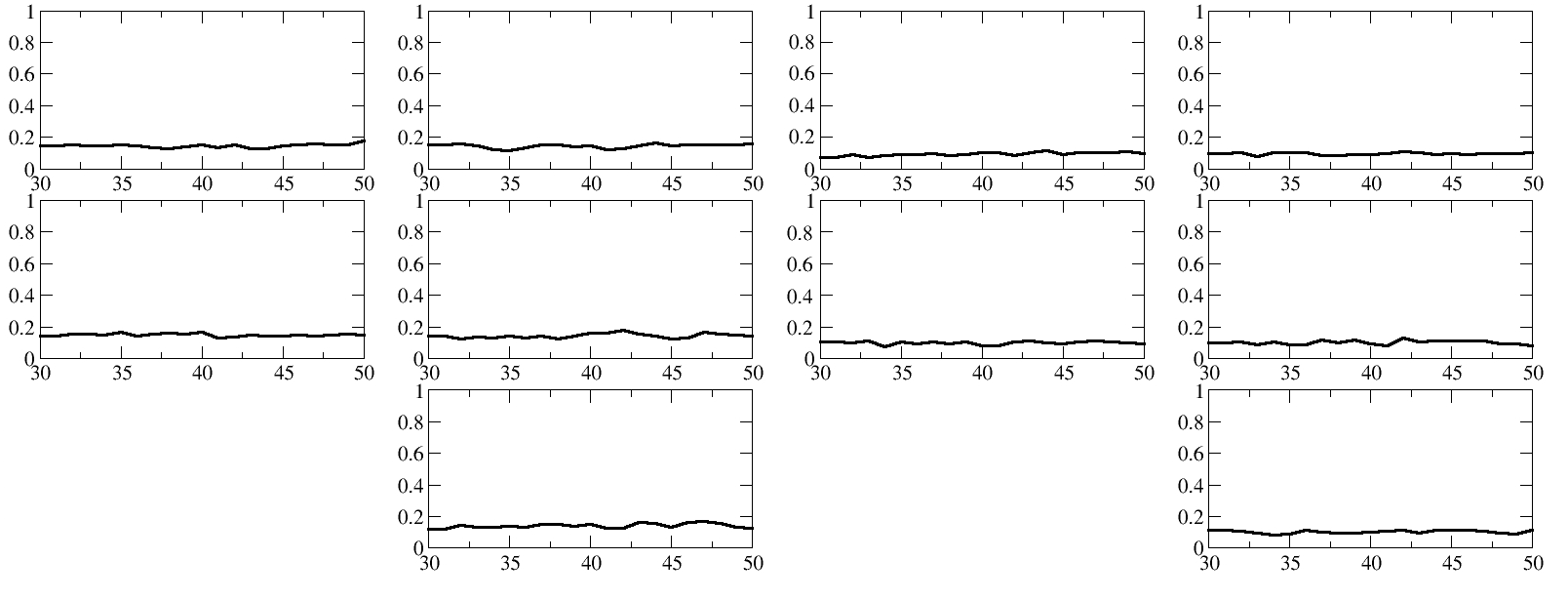


**Table S1** Average number of hydrogen bonds between the backbone peptide C=O of residues *i* and the backbone peptide NH of the residues *i + 4* and average total number of hydrogen bonds within sequence over final 20 ns of REMD for sequences **WT**, **I**, **R** and **RI**. Standard deviations in parentheses.

| ***i → i+4*** | ***i(WT/I)*** | ***i(R/RI)*** | **WT** | **I** | **R** | **RI** |
| --- | --- | --- | --- | --- | --- | --- |
| 0 – 4 | Ace | Ace | 0.04 (0.19) | 0.04 (0.19) | 0.00 (0.02) | 0.00 (0.03) |
| 1 – 5 | Ser | Asn | 0.04 (0.21) | 0.04 (0.20) | 0.09 (0.28) | 0.05 (0.22) |
| 2 – 6 | Gln | Glu | 0.11 (0.31) | 0.10 (0.30) | 0.31 (0.46) | 0.30 (0.46) |
| 3 – 7 | Glu | Pro | 0.08 (0.26) | 0.07 (0.25) | 0.18 (0.39) | 0.17 (0.38) |
| 4 – 8 | Thr | Leu | 0.14 (0.34) | 0.12 (0.33) | 0.26 (0.44) | 0.23 (0.42) |
| 5 – 9 | Phe | Leu | 0.16 (0.37) | 0.18 (0.39) | 0.28 (0.45) | 0.28 (0.45) |
| 6 – 10 | Ser | Lys | 0.05 (0.22) | 0.06 (0.23) | 0.12 (0.33) | 0.11 (0.32) |
| 7 – 11 | Asp | Trp | 0.05 (0.21) | 0.04 (0.20) | 0.16 (0.37) | 0.14 (0.35) |
| 8 – 12 | Leu | Leu | 0.25 (0.43) | 0.26 (0.44) | 0.15 (0.36) | 0.14 (0.35) |
| 9 – 13 | Trp | Asp | **-** | **-** | 0.05 (0.22) | 0.06 (0.23) |
| 10 – 14 | Lys | Ser | 0.00 (0.00) | 0.00 (0.01) | 0.09 (0.29) | 0.11 (0.31) |
| 11 – 15 | Leu | Phe | 0.02 (0.15) | 0.01 (0.12) | 0.15 (0.36) | 0.14 (0.35) |
| *total* |  |  | 0.94 (0.92) | 0.93 (0.91) | 1.84 (1.48) | 1.74 (1.45) |
